# Lightweight physiologic sensor performance during pre-hospital care delivered by ambulance clinicians

**DOI:** 10.1007/s10877-015-9673-z

**Published:** 2015-03-25

**Authors:** Alasdair J. Mort, David Fitzpatrick, Philip M. J. Wilson, Chris Mellish, Anne Schneider

**Affiliations:** 1 dot.rural Digital Economy Hub, King’s College, University of Aberdeen, Aberdeen, Scotland, UK; 2 The Centre for Rural Health, University of Aberdeen, Centre for Health Science, Old Perth Road, Inverness, Scotland, UK; 3 Scottish Ambulance Service, Gyle Square, Edinburgh, Scotland, UK; 4 Department of Computing Science, King’s College, University of Aberdeen, Aberdeen, Scotland, UK

**Keywords:** Physiologic monitoring, Pre-hospital, Ambulance clinicians, Motion artefact

## Abstract

The aim of this study was to explore the impact of motion generated by ambulance patient management on the performance of two lightweight physiologic sensors. Two physiologic sensors were applied to pre-hospital patients. The first was the Contec Medical Systems CMS50FW finger pulse oximeter, monitoring heart rate (HR) and blood oxygen saturation (SpO2). The second was the RESpeck respiratory rate (RR) sensor, which was wireless-enabled with a Bluetooth^®^ Low Energy protocol. Sensor data were recorded from 16 pre-hospital patients, who were monitored for 21.2 ± 9.8 min, on average. Some form of error was identified on almost every HR and SpO_2_ trace. However, the mean proportion of each trace exhibiting error was <10 % (range <1–50 % for individual patients). There appeared to be no overt impact of the gross motion associated with road ambulance transit on the incidence of HR or SpO_2_ error. The RESpeck RR sensor delivered an average of 4.2 (±2.2) validated breaths per minute, but did not produce any validated breaths during the gross motion of ambulance transit as its pre-defined motion threshold was exceeded. However, this was many more data points than could be achieved using traditional manual assessment of RR. Error was identified on a majority of pre-hospital physiologic signals, which emphasised the need to ensure consistent sensor attachment in this unstable and unpredictable environment, and in developing intelligent methods of screening out such error.

## Introduction

The pre-hospital context is a notoriously difficult environment in which to measure patient physiology accurately and reliably. If the patient is trapped (e.g. following a road traffic collision) then it may be difficult to make appropriate manual assessments or to apply electronic monitoring equipment. There is often movement of unpredictable amplitude and acceleration in multiple directions. For example, the patient may be moved in the process of immediate, potentially life-saving management. This might include clearing the patient’s airway, inserting a device that protects the airway, conducting chest compressions where the patient is in cardiac arrest, or moving an unconscious but breathing patient into the recovery position.

Patients must also be moved to a location where they can receive definitive treatment for their illness and/or injury. However, it may take more than one journey and there may be intercurrent treatment at more than one site before definitive care is reached. In most cases pre-hospital patients are transported by emergency ambulance, which in the United Kingdom are staffed by a mixture of qualified Paramedics and Technicians; those qualified to a lower level than Paramedics with a smaller skill-set. The time taken to transport patients to hospital can vary greatly, influenced mainly by the geographic site of the emergency. That is, it can take much longer to transport rural patients to hospital than it might do in urban centres. Much of the patient assessment and physiologic monitoring conducted by rural ambulance clinicians thus takes place during road and air transit.

Standard physiologic parameters (e.g. blood pressure, heart rate, respiratory rate) play a key role in the triage of pre-hospital patients as they may indicate present and future patient deterioration. For example, validated systems such as the National Early Warning Score are now widely used [1]. Hillman et al. [2] reported serious physiological abnormalities in 29 % of patients in the 8 h prior to death (excluding cardiac arrests and deaths whilst in intensive care). Also, one-third of patients who did not have ‘do not resuscitate’ orders had persistently abnormal physiology for 2 days prior to death. The physiologic abnormality reported most often was hypotension, followed by tachypnoea, then tachycardia. Other studies have also reported considerable instability in standard physiologic measures prior to a major, life-threatening event (e.g. respiratory arrest, cardiac arrest, haemorrhagic hypotension) [3–5], although this is not always the case [6, 7].

The physiologic monitoring systems operated by ambulance clinicians most often take the form of a single device that measures several parameters. For example, the Scottish Ambulance Service operated the Philips HeartStart MRx system (Philips, Netherlands), which apart from being a defibrillator measured blood pressure on the upper-arm, and blood oxygen saturation and heart rate through a pulse oximeter attached to a finger. The monitor also captured a 12-lead ECG, and could be linked via Bluetooth^®^ to a mobile phone from where the ECG was transmitted to a coronary care unit for expert advice. Monitors like these are suitable for ambulance use as they are rugged and can be removed from the vehicle to conduct monitoring where a patient is located (e.g. in their house, by the roadside). However, they tend to be relatively heavy (due to battery requirements) and the sensors are wired. Wires can be snagged, pulling on the site of attachment resulting in spurious readings and even pulling the sensor(s) off the patient altogether. There have also been anecdotal reports of emergency workers accidentally cutting cables during the extrication process. Such systems provide continuous monitoring, although the recording of individual values to care provider files is usually performed manually, limiting the volume of data recorded to perhaps two or three data points (depending upon the duration of patient transport). There is evidence that having a much larger volume of physiologic data is a better predictor of later mortality than relying upon a single value [8].

Lightweight wireless physiologic monitors are now in existence, developed partly through an international effort to enable and enhance the monitoring of patients in their own homes (i.e. telehealth). We propose that such monitors could play a major role in the future of pre-hospital care, employed by ambulance clinicians where lightweight, wireless monitoring could convey an advantage over current heavy, wired systems. We also contend that these monitors may be beneficial to Community First Responders (CFRs) who volunteer to deliver basic first-aid for ambulance services whilst an emergency ambulance is on its way. Such devices, if simple to apply and use, could greatly increase the volume of data that such personnel are able to capture, and potentially alert them to patient deterioration that they may be able to address within their limited skill-set. Facilitating the capture of more physiologic data is particularly important given the general lack of evidence to support CFR activity; this would help to inform their practice and policy. However, this is only applicable if such technology can deliver accurate data reliably during the unstable and unpredictable context of pre-hospital care.

Commonly-recorded physiologic parameters are influenced by motion and in turn display artefact or ‘noise’; physiologic waveforms deviate from their ‘normal’, characteristic patterns and erroneous data are potentially reported to the user. Such error could take the form of falsely low or high readings, often triggering alarms that then distract the operator from keeping a physical watch on the patient. Most physiologic sensors include some form of signal processing so that they can continue to deliver data in the face of motion or extreme physiologic compromise. However, it is reasonable to postulate that there is a threshold beyond which a physiologic sensor will no longer be able to deliver accurate data; for example, if finger perfusion is so low that it is impossible to generate valid blood oxygen saturation and pulse data.

The aim of our study was to explore the impact of ambulance clinician patient management and transport on defined parameters recorded from patients by two lightweight physiologic sensors. This contributed to the University of Aberdeen Managing Information in Medical Emergencies (MIME) project (see www.dotrural.ac.uk/mime), which developed and evaluated technology to support CFRs at the scene of rural medical emergencies [9].

## Materials and methods

### Design

The study employed a ‘field-function’ design, which involved controlled ‘pseudo-deployment’ of the physiologic sensors in a real-life situation [10].

### Setting

**Fig. 1:**
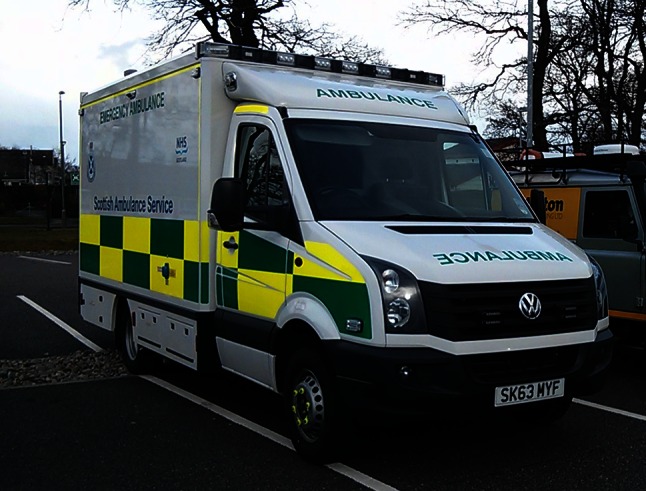
Scottish Ambulance Service emergency vehicle

The study took place at a Scottish Ambulance Service station in the north of Scotland, UK (Fig. 1). The station responded to a variety of types of call-outs originating from urban and rural areas.

### Participant identification and recruitment

#### Ambulance clinicians

Clinicians from two station ‘watches’ were invited to take part. Clinicians attended an evening presentation at which the study and their proposed role in the research were described. All clinicians who volunteered to take part were asked to provide written, informed consent.

#### Pre-hospital patients

The first approach to patients was made by recruited ambulance clinicians according to set inclusion criteria (Table 1). The decision of whether or not to approach each patient was made ultimately at clinicians’ discretion. They were only to approach patients if the application of the physiologic sensors did not interfere with the timely delivery of their ‘usual’ care.

**Table 1:** Pre-hospital patient inclusion and exclusion criteria

Inclusion criteria	Exclusion criteria
Adults (18 years and above)	Unable to understand verbal explanations given in English—also including those with special communication needs
Males and females	Patients with injuries or in a position that prevented application of the sensors
Ability to represent own interests and to provide verbal, informed consent	
Able to apply the sensors to the patient	

The first stage of consent involved clinicians describing the study to patients verbally. Patients were given the opportunity to ask any questions, and if they were happy to proceed they provided verbal informed consent on-scene. A preliminary verbal informed consent was deemed appropriate considering the relatively low risk nature of the study. The second stage of consent involved sending patients an opt-out form to their home address within a study information pack, at least 2 weeks after their contact with the research. Whilst consent mechanisms based on opting out are not the norm, these have previously been carried out in other pre-hospital emergency care research where patients were in a vulnerable state immediately after their emergency care visit, and are well recognised as being a difficult group to make contact with [11, 12]. We considered opt-out consent to be satisfactory in this situation where there was no significant risk of harm to participants and no risk to patient confidentiality. An opt-in approach could have resulted in lower recruitment and therefore lower generalisability of the results [13].

The local NHS Health Board was contacted prior to mailing study information packs in order to establish whether or not patients had been discharged from hospital. Patients who had died and those who completed the opt-out form were excluded from the study. All patients who participated were given a unique identification number in order to anonymise their involvement.

### Physiologic parameters

Three physiologic parameters were selected to monitor; respiratory rate (RR), heart rate (HR) and blood oxygen saturation (SpO_2_). RR was chosen as it is an essential clinical parameter that traditionally is difficult to monitor both reliably and repeatedly in anything but a resting, motionless patient. Indeed, RR has previously been described as the ‘neglected vital sign’ [14]. In the pre-hospital environment, ambulance clinicians will monitor RR by counting the rise and fall of the patient’s chest/abdomen and/or the misting and de-misting of a non-rebreathing oxygen mask (not including RR monitored using capnography in the intubated patient). This means that only a small number of RR data points are recorded during the time that ambulance clinicians are with the patient. HR and SpO_2_ were selected as they are also parameters that are monitored ubiquitously in the pre-hospital environment using pulse oximetry.

Respiratory rate, HR and SpO_2_ formed the basis of a novel pre-hospital physiologic monitoring system that we developed within our research group for use by Ambulance Service CFRs. They are all parameters that can be monitored using lightweight physiologic sensors that are simple and quick to apply by non-medical experts.

### Physiologic sensors

Two lightweight, non-invasive physiologic sensors were selected for application to ambulance patients. The first was the Contec Medical Systems CMS50FW pulse oximeter (Contec Medical Systems, Qinhuangdao, China), which monitored SpO_2_, HR and also displayed the photoplethysmograph to the user.

The CMS50FW had Bluetooth^®^ capability to send data wirelessly. However, this facility was turned off in this study and the data stored on the device instead (NOT including photoplethysmograph data). Capturing data wirelessly would have necessitated a separate laptop computer and time-consuming device pairing, both of which were inappropriate in the space-restricted and time-dependent emergency ambulance environment. Non-averaged HR and SpO_2_ data were recorded in a comma separated value file at a rate of 1 Hz.

**Fig. 2:**
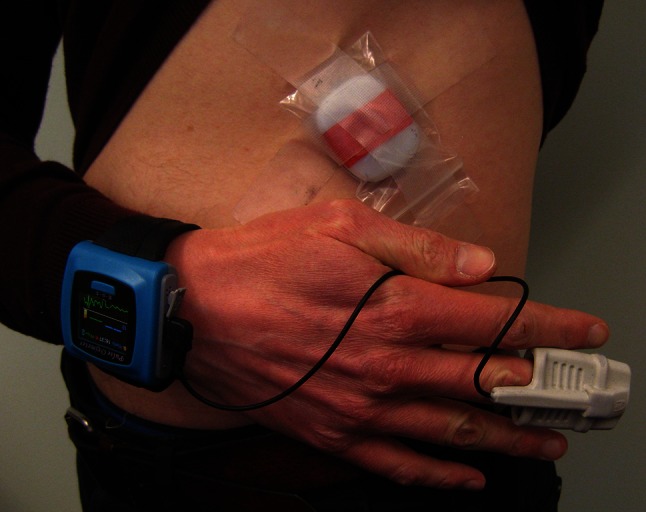
Medical sensors employed in the pre-hospital fieldwork

The second monitor was the RESpeck RR sensor (University of Edinburgh Department of Speckled Computing, School of Informatics, Scotland), which was an encapsulated tri-axial accelerometer positioned on the left side of the abdomen just under the costal margin. RESpeck recorded changes in abdominal position in three orthogonal axes, relative to gravity. These data were automatically integrated and differentiated into a derived ‘activity’ signal, and a RR signal with a shape similar to inspiratory and expiratory flow. RESpeck had been verified to be a reliable measure of RR when compared with RR derived from a nasal cannula in anaesthetised post-operative patients; instantaneous RESpeck RR matched the routine clinical measurement of RR within two breaths per minute (bpm)—an acceptable limit of accuracy employed previously—on 86 % of occasions, with a mean absolute difference of 0.6 bpm [15]. However, our study was the first time that RESpeck had been implemented in the pre-hospital environment. RESpeck was entirely wireless and all data were transmitted using a Bluetooth^®^ 4.0 Low-Energy protocol to an iPod (Apple, Cupertino, CA, USA) on which the data were displayed and recorded. Accelerometer data were recorded at a rate of 12.5 Hz. Figure 2 displays both medical sensors and their method of attachment to the body.

### Study protocol

Upon arrival on-scene, ambulance clinicians approached each patient and carried out a primary survey of their clinical status. If the patient provided verbal informed consent the aim was to apply the sensors as early as appropriate. The pulse oximeter was attached first by ambulance clinicians to patients’ index fingers. A stopwatch was started at the point that pulse oximeter data recording was initiated, which provided a ‘time zero’ reference. Secondly, the RESpeck RR sensor was enclosed in a protective plastic sleeve (to meet infection control requirements) and attached to the abdomen using Transpore™ medical tape (3 M Healthcare, USA). It was then paired with the iPod and data recording commenced 30 s after the pulse oximeter data stream began. Data were recorded from both sensors until the end of ambulance clinician management, which was most often at the point of handover to hospital Emergency Department staff. However, there were a small number of occasions where patients were transported directly by ambulance to a receiving ward (i.e., for referrals by General Practitioners), or were not transported by ambulance at all if it was deemed appropriate to leave them at home.

A researcher (AM) travelled as an observer in the ambulance, and only came into contact with patients if they had provided verbal consent. The researcher oversaw the application of the sensors by ambulance clinicians and was responsible for starting and stopping data recording on each device. He also carried a Getac Z710 rugged tablet computer (Getac, Irvine, CA, USA) that ran software (University of Aberdeen) that captured the input of contextual information about patient management and clinical status. This was essential in order to explore the effect of patient management on sensor data. A copy of the electronic Patient Report Form (ePRF) was retrieved for each patient in order to gather as much contextual data about each patient and their management as possible. The ePRF contained all clinical data, including interventions, recorded by ambulance clinicians. The form permitted the formal handover of information between ambulance clinician and Emergency Department staff on arrival at hospital. Each record contained its own unique incident number that enabled calls to be traced and patients identified at a later date if necessary.

Data collection proceeded until reasonable “saturation” (defined as the point at which no new patterns of data were emerging) was achieved, assessed by author AM. This approach to sampling is commonly used in qualitative research and was appropriate in this exploratory study.

### Data analysis

#### Pulse oximetry

Heart rate and SpO_2_ data were initially reviewed and plotted to explore for any gross deviations from ‘normal’ physiologic values. In particular, the pulse oximeter logged a non-physiologic value of 255 BPM for HR and 127 % for SpO_2_ when the sensor was removed from the finger, or if finger attachment was sub-optimal. The frequency of such values was noted. The maximum, minimum, mean and standard deviation for HR and SpO_2_ were then calculated on a patient-by-patient basis. Further analyses explored for the presence and length of any periods of pulse oximeter data that appeared ‘abnormal’. Our definition of ‘abnormal’ also included any incidence of a sudden, apparently non-physiologic, increase or decrease in blood oxygen saturation and/or heart rate from a stable value. The total time that each sensor exhibited ‘abnormality’ was expressed as a percentage of the total monitoring time. This was also expressed as the proportion of ‘abnormality’ at nominal rest, and the proportion of ‘abnormality’ during ambulance transit, in order to explore for any apparent impact of gross motion on signal quality.

#### Respiratory rate

RESpeck files were downloaded from the iPod and the gross activity and breathing signals were plotted. Patient management data were plotted on top of each trace, noting in particular the start and end of gross motion associated with ambulance transit. The raw data files were then processed post hoc using proprietary software (University of Edinburgh), which produced a separate file containing time and validated RR data. The software actively excluded any data captured in excess of a pre-defined movement threshold. This meant that only the fine movements associated with breathing were analysed, and that larger movements not associated with breathing were omitted. The number of validated breaths captured before, during and after ambulance transit were noted. The maximum, minimum and mean number of validated breaths captured before and after ambulance transit per minute was also calculated; zero data (i.e. where no validated breaths were produced per minute) were included in the mean data. Finally, maximum, minimum, and the mean (±1 SD) RR were recorded.

### Ethical approval

The study was approved by an NHS Research Ethics Committee and by the Scottish Ambulance Service’s Research Governance Group.

## Results

### Patients

A total of 20 pre-hospital patients gave verbal consent to take part in the study. Data for four patients were excluded: three patients opted out, and one patient died sometime after admission to hospital. This left a total of 16 patient data sets for inclusion (ten male, six female; age range 42–96 years). Patients were managed by ambulance clinicians for a wide variety of suspected medical problems and injuries (Table 2); there were 12 emergency calls, three urgent call-outs (requested by local General Practitioners) and one patient transfer from hospital to a local airport. A majority of patients (n = 13) were transported to the local hospital (10 to the Emergency Department and three to an acute receiving ward). Two patients (both emergency calls) were not transported to the Emergency Department; one was a diabetic whose blood sugar levels returned to normal after treatment, and the other was a bariatric patient who had fallen but on assessment did not have any injury or illness.

**Table 2:** Pre-hospital patient clinical status

Patient ID PM—male PF—female	Working clinical assessment	Response	Airway	Breathing rate range (breaths per min)	Pulse rate range (beats per min)	SpO_2_ range (%)	Glasgow coma scale
PM1	Central chest pain	Alert	Clear	14–20	46–62	99–100	15
PM3	Road traffic collision injuries (some pain, abrasions and contusions)	Alert	Clear	16	74–76	98	15
PM4	Chest pain	Alert	Clear	16	97–105	97–100	15
PM5	NA—transfer from hospital to airport after discharge	Alert	Clear	16–17	73–74	93–95	15
PM7	Non-traumatic back pain	Alert	Clear	16–24	80–100	94–99	15
PM8	Unknown problem	Alert	Clear	16	106	100	15
PM9	Diabetic	Alert	Clear	16	70–75	93–96	15
PM10	Sick person	Alert	Clear	12–14	62–65	94	14
PM11	Diabetic	Alert	Clear	14–16	100–110	95–97	15
PM12	Fall	Alert	Clear	15	114	92	15
PF1	Fall	Alert	Clear	Not recorded	81	98	15
PF2	Back pain	Alert	Clear	16–24	70–88	98–99	15
PF3	Sick person	Alert	Clear	20	101	100	15
PF6	Stroke, numbness, paralysis, or movement problems	Responding to pain	Clear	15	70	95	14–15
PF7	Abdominal pain	Alert	Clear	32	74	99	14
PF8	Stroke history	Alert	Clear	19	64	98	15

NB these are the clinical working assessments recorded by ambulance clinicians immediately prior to ending their management

### Monitoring time

Mean sensor monitoring time was 21.2 ± 9.8 min (total = 5.7 h; range 7.4–41.5 min). For patients who were transported to hospital, mean monitoring time at nominal rest (i.e. before and after gross motion associated with ambulance transit) was 11.4 ± 9 min (range 3.3–36.8 min), and mean road transport time was 12.9 ± 8.2 min (range 3–27.4 min).

### Heart rate and blood oxygen saturation

The pulse oximeter was applied successfully to all patients. Mean HR was 83.7 ± 10.5 BPM (maximum = 166 BPM, minimum = 49 BPM). Mean SpO_2_ was 93.9 ± 0.9 % (maximum = 99 %, minimum = 79 %). Non-physiologic, ‘abnormal’ values and signal patterns were present on a majority of HR and SpO_2_ traces (n = 12/16 for HR, n = 13/16 for SpO_2_). HR traces mirrored the ‘abnormalities’ seen on SpO_2_ traces, and vice versa, on all but one occasion.

#### Heart rate

**Fig. 3:**
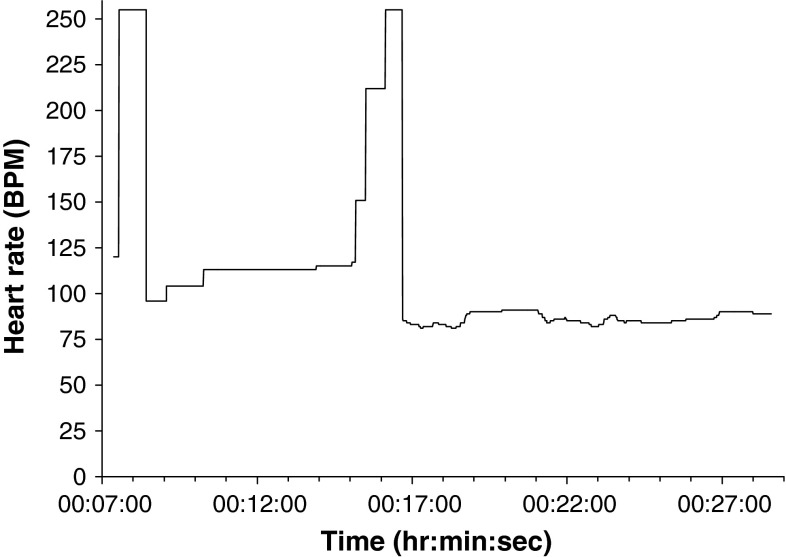
Example of pulse oximeter HR abnormality (Patient PM7)

The most frequent non-physiologic HR value returned was 255 BPM (n = 12/16), which was what the sensor recorded in its memory when it was removed from the finger. However, it was apparent from observing the monitoring process that the pulse oximeter was removed from patients’ fingers in the middle of monitoring on only two occasions. Hence, 255 BPM was recorded when the pulse oximeter monitoring conditions were sub-optimal, whilst the sensor was still attached to the finger. The other type of apparent error noted (n = 3/16) was rapid, non-physiologic drops and increases in HR, with periods in-between where HR remained artificially static. This was contrary to the normal physiologic undulations in HR that were evident in other parts of the trace for the same patient (Fig. 3).

The absolute duration of individual HR errors ranged from 2 s up to almost 20 min in the most extreme case (mean 80.9 ± 237.5 s). HR error occurred between one and five times for each patient. The proportion of each HR trace that exhibited apparent error ranged from under 1 to almost 50 % (mean 8.8 ± 13.9 %); however, the proportion of error was <10 % on 9/12 occasions, and <5 % on 7/12 occasions, where error presented. There appeared to be no effect of gross motion associated with ambulance transit on the incidence of HR error, or on the duration of individual HR errors.

#### Blood oxygen saturation

**Fig. 4:**
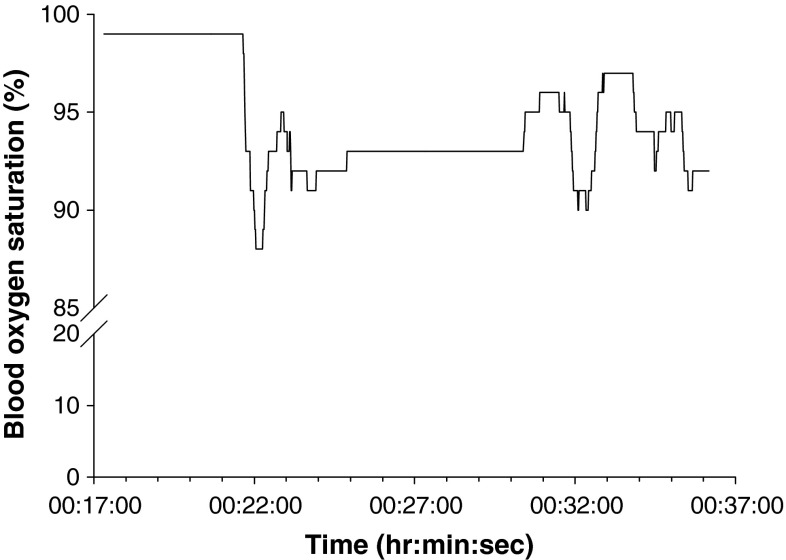
Example of SpO_2_ apparent error (Patient PM7)

**Fig. 5:**
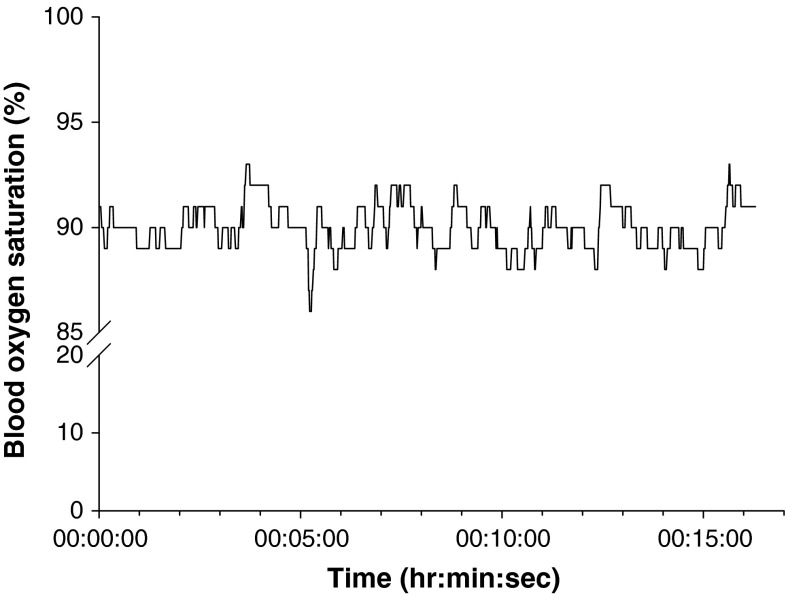
Example of ‘normal’ SpO_2_ fluctuation (Patient PF3)

The non-physiologic SpO_2_ value recorded most frequently was 127 % (n = 12/16). SpO_2_ traces also exhibited fluctuations in the form rapid drops and rises, a minority of which appeared to be error (n = 3/16) (Fig. 4), whilst others appeared physiologically feasible (Fig. 5). SpO_2_ was more stable for some patients, and less so for others.

The range of duration of individual SpO_2_ errors was almost exactly the same as for HR, with each error occurring between one and four times for each patient. The proportion of each SpO_2_ trace that included apparent error ranged from under 1 % to almost 50 % (mean 7.6 ± 13.2 %); 10/13 traces <10 %, and 8/13 traces <5 %. Just as for HR, there appeared to be no effect of gross motion associated with ambulance transit on the incidence of SpO_2_ error, or on the duration of individual errors. HR and SpO_2_ errors tended to occur at the same time.

### Respiratory rate

The RESpeck sensor was applied successfully to 14/16 patients. On two occasions the patient’s clothing impeded application to the abdomen. On average, 40 % of activity data were below the pre-defined activity threshold, and 60 % (±18.9, 1 SD) were above it. This meant that 60 % of data were actively excluded from the RESpeck post hoc analysis. However, the proportion of data in excess of the activity threshold ranged between 27.4 and 86.5 % in individual patients, meaning that the level of motion varied widely.

The total number of validated breaths captured at nominal rest (i.e., without the gross motion associated with ambulance transit) during individual patient management ranged from 5 to 255, with an average of 54.6 breaths captured per patient (±65, 1 SD). The maximum number of validated breaths returned by RESpeck for each patient ranged between 3 and 18 breaths per minute. The mean number of validated breaths reported per minute, including zero values for minutes where no validated breaths were returned, ranged from 0.5 to 7.9 (overall mean across all patient data = 4.2 ± 2.2, 1 SD). Breathing rate ranged from 5.3 to 35.3 breaths per minute, whilst mean breathing rate for each patient ranged from 9.7 to 21.2 breaths per minute.

Only 29 validated breaths were captured during ambulance transit across 3/11 patients (n = 15, 12, 2), compared to 765 breaths recorded at nominal rest from 14 patients.

## Discussion

This was the first study of its kind to robustly measure the impact of pre-hospital motion on commonly monitored physiologic parameters. Our research study identified some form of error in nearly every blood oxygen saturation, heart rate and breathing rate signal. For most pre-hospital patients error accounted for a relatively small proportion of pulse oximeter recording. The breathing rate sensor delivered considerably less data, but still produced many more data points than would be achievable through manual assessment alone.

Signal artefact is a key limitation of pulse oximeter technology, which can arise from low signal-to-noise ratio and from false signals [16]. Pulse oximetry relies on the assumption that all of the pulsating blood is arterial. However, motion mobilises venous blood, which has a lower SpO_2_ and mixes with the arterial component. Motion thus tends to lower SpO_2_ and produce false alarms [17]. This effect is exaggerated if there is low perfusion to the site of monitoring. Clinical studies have demonstrated this effect. For example, Wikilund et al. [18] noted that, post-anaesthesia, the pulse oximeter alarmed every eight minutes, on average. Some 77 % of oximeter alarms were found to be false, with motion indicated as one contributing factor. It also appeared that finger pulse oximeters demonstrated a poorer true/false ratio than ear pulse oximeters (18 vs. 29 %). Tsien and Fackler [19] reported that >90 % of SpO_2_ and heart rate alarms generated by pulse oximeters in an intensive care unit were false positives. Generally speaking, pulse oximeters result in more false-positive alarms than other physiologic monitoring systems. The clinically-relevant positive predictive value (=number of clinically-relevant true positives divided by clinically-relevant true positives + clinically-irrelevant true positives + false positives) for pulse oximetry has, accordingly, been reported to be very low (≤6 %, for both SpO_2_ and the derived heart rate value, Ibid). However, it is important to note that the threshold of alarms will vary from study to study, and the threshold is open to adjustment by users.

Langton and Hanning studied the ability of four different pulse oximeters to identify simulated hypoxaemia in healthy volunteers during two levels of controlled vibration (sine wave 4 Hz and intermittent 8 Hz; the 8 Hz condition was representative of the motion experienced during patient transport) [20]. The vibration sometimes resulted in false decreases in SpO_2_ in 3/4 oximeters, which was similar to the current study, but was not identical as such patterns were not identified across all of our 16 patients. Vibration also lengthened the time taken for the pulse oximeters to detect hypoxaemia. There were also differences between the individual pulse oximeters under test, reflecting the varying capacities of the different algorithms to deal with motion.

Perhaps the seminal piece of research on characterising motion in a very wide variety of clinical environments was that conducted by Tobin et al. [21]. Some 350 patients were monitored, of whom 70 exhibited motion (20 %); 35/70 moving patients were instrumented for detailed analysis. This included three patients who had been transported by ambulance. Ambulance transit at reasonably high speed (60 mph) resulted in a very noisy pulse oximeter signal; indeed one of the largest in the study’s clinical cohort. However, the investigators did note that the magnitude of disturbance to the underlying photoplethysmograph was not directly related to the absolute force of movement. For example, a patient flexing their foot resulted in more oximeter signal deformation than that caused when the leg twitched. This indicated that there were underlying, vascular mechanisms at play. One of the difficulties in studying pulse oximetry during the gross motion of ambulance transit is that it is difficult to ascertain just how much error is due to vehicle motion, and how much is due to the patient moving the site of monitoring; using an accelerometer as a reference sensor may be an appropriate solution. Silbergleit et al. [22] attempted to quantify the forces experienced during road ambulance transport at 35 mph. They identified that road ambulance vibration varied greatly—generally occurring <1 Hz and from 10 to 15 Hz—and was highest in the inferior and superior axes. The largest peak accelerations were also in the inferior and superior planes (0.8 and 0.7 g respectively).

This was the first application of the RESpeck breathing rate sensor in a pre-hospital context. However, no validated breathing rates were delivered by the sensor during ambulance transit; the fine excursions of the abdomen with breathing were lost amongst the large, random movements of the ambulance. Also, even the entry and exit of clinicians into and from the ambulance (without the patient) was sufficient for it not to deliver validated data. Despite this, only reporting on rhythmic, regular breaths is more diagnostic than reporting on irregular, noisy breathing rate data. For example, Chen et al. [23] employed the technique of impedance pneumography (measuring the changes in resistance across the chest with breathing, using a conventional electrocardiogram trace) combined with a novel algorithm (applied retrospectively) on 898 trauma patients monitored during helicopter transit. Breathing rate based upon reliable breaths only was a better predictor of a patient receiving a respiratory intervention at a later stage, and of identifying patients with haemorrhage. Impedance pneumography (available on some pre-hospital multi-parameter monitoring systems) will inevitably capture more data on respiratory rate than the RESpeck during gross motion. However, our proposed model of employing the RESpeck on pre-hospital patients managed by Community First Responders will not mean exposure to ambulance transit. As such, the RESpeck may be an effective way for non-experts to gather considerably more breathing rate data than would be achievable using manual methods alone. It may also be more objective.

There were some limitations to this study. Pulse oximeter photoplethysmograph data were displayed on the screen of the commercially-available pulse oximeter, but the device did not permit recording of the raw photoplethysmograph to file in ‘non-wireless’ mode. Wireless data transfer for the pulse oximeter under test was time-consuming and necessitated a separate laptop computer; there was neither sufficient time nor space to effect this in the emergency ambulance. This prevented any detailed analysis of the impact of motion on the underlying signal (e.g. motion artefact issues during driving). However, some data to this effect have been reported previously [21]. Our next study will be a ‘laboratory-function’ experiment where there will be more time and space to conduct wireless data transfer and capture raw data. A single pulse oximeter was tested in this study; it is likely that other sensors employ different algorithms and thus respond to motion differently. The study was also a proxy for the proposed context of employing the sensors in our MIME system; i.e. patients managed by ambulance clinicians, and not Community First Responders. This meant that the motion that the sensors was exposed to was much greater, although the study did still include periods of reduced motion before and after ambulance transit. However, conducting the study in emergency ambulances was the quickest and safest method for collecting our data; first responders see relatively few patients and have limited first-aid training. Finally, the study only included patients who did not have immediately life-threatening medical conditions or trauma (i.e. only those who were able to provide verbal informed consent).

To conclude, this study identified that all of the physiologic sensors exhibited some error on almost every patient recording during ambulance clinician patient management. However, this accounted for a relatively small proportion of the total monitoring time, on average. Error mostly took the form of non-physiological blood oxygen saturation and heart rate values, and rapid step changes often to much lower values. The RESpeck breathing rate sensor did not exhibit error per se. Rather, if the pre-defined threshold of motion was crossed it did not deliver breathing rate data, which was as it was designed to do. It was very positive that many more validated breathing rate points were achieved through employing RESpeck than via manual assessment. The almost complete lack of validated breathing rate data during ambulance transit was inconvenient, but would not be problematic in our proposed model of use by CFRs (i.e. where there is no ambulance transit).

Future work in this area should focus on sensor-produced breathing rate data during motion, which is currently not recorded during ambulance transit. The development of a single sensor to monitor all three parameters (RR, HR and SpO_2_) would also be valuable, minimising the time taken to apply equipment and simplifying the process for non-expert users such as CFRs.
